# Development of a Nanobody-Based Competitive Enzyme-Linked Immunosorbent Assay for Efficiently and Specifically Detecting Antibodies against Genotype 2 Porcine Reproductive and Respiratory Syndrome Viruses

**DOI:** 10.1128/JCM.01580-21

**Published:** 2021-11-18

**Authors:** Hong Duan, Xu Chen, Jiakai Zhao, Jiahong Zhu, Guixi Zhang, Mengnan Fan, Beibei Zhang, Xueting Wang, Yani Sun, Baoyuan Liu, En-Min Zhou, Qin Zhao

**Affiliations:** a Department of Preventive Veterinary Medicine, College of Veterinary Medicine, Northwest A&F University, Yangling, Shaanxi, China; b Scientific Observing and Experimental Station of Veterinary Pharmacology and Diagnostic Technology, Ministry of Agriculture, Shaanxi, China; Mayo Clinic

**Keywords:** nanobody, nanobody-HRP fusion protein, HEK293S cells, competitive ELISA, genotype 2 PRRSV

## Abstract

Porcine reproductive and respiratory syndrome virus (PRRSV) infection causes considerable economic loss to the global pig industry. Efficient detection assay is very important for the prevention of the virus infection. Nanobodies are the advantages of small molecular weight, simple genetic engineering, and low production cost for promising diagnostic application. In this study, to develop a nanobody-based competitive ELISA (cELISA) for specifically detecting antibodies against PRRSV, three nanobodies against PRRSV-N protein were screened by camel immunization, library construction, and phage display. Subsequently, a recombinant HEK293S cell line stably secreting nanobody-horseradish peroxidase (HRP) fusion protein against PRRSV-N protein was successfully constructed using the lentivirus transduction assay. Using the cell lines, the fusion protein was easily produced. Then, a novel cELISA was developed using the nanobody-HRP fusion protein for detecting antibodies against PRRSV in pig sera, exhibiting a cut-off value of 23.19% and good sensitivity, specificity, and reproducibility. Importantly, the cELISA specifically detect anti-genotype 2 PRRSV antibodies. The cELISA showed more sensitive than the commercial IDEXX ELISA kit by detecting the sequential sera from the challenged pigs. The compliance rate of cELISA with the commercial IDEXX ELISA kit was 96.4%. In addition, the commercial IDEXX ELISA kit can be combined with the developed cELISA for the differential detection of antibodies against genotype 1 and 2 PRRSV in pig sera. Collectively, the developed nanobody-based cELISA showed advantages of simple operation and low production cost and can be as an assay for epidemiological investigation of genotype 2 PRRSV infection in pigs and evaluation after vaccination.

## INTRODUCTION

Porcine reproductive and respiratory syndrome (PRRS), caused by PRRS virus (PRRSV), is a major disease threatening large-scale pig farms and is characterized by reproductive disorders in sows and respiratory diseases of pigs of all ages, especially piglets (1). Currently, PRRSV continues to cause considerable economic loss to the global pig industry; specifically, the United States loses approximately US$600 million annually due to the virus (2). PRRSV is an enveloped RNA virus, positive-stranded, belonging to the genus *Arterivirus*, family Arteriviridae, order *Nidovirales* (3). It contains at least 10 open reading frames (ORFs), including ORF1a, ORF1b, ORF2a, ORF2b, ORF3, ORF4, ORF5, ORF5a, ORF6, and ORF7 (4, 5). PRRSV is divided into two types based on genetic distances: genotype 1 (European) and genotype 2 (North American) (6, 7). Particularly, the genotype 2 PRRSV strains are the predominant pathogens that cause clinical outbreaks of PRRS in North America and China (8). However, the genotype 1 PRRSV has attracted increasing attention, and multiple strains have been recently isolated and identified in China (8, 9). These two genotypes share only approximately 60% nucleotide identities and do not produce cross-protection (10).

The capsid protein of PRRSV (PRRSV-N protein) encoded by ORF7 gene is relatively conserved and accounts for 20%–40% of the total amount of viral particle. It has good antigenicity and immunogenicity, and anti-PRRSV-N protein antibodies can be detected at 7 days postinfection (11, 12). Therefore, the PRRSV-N protein is an ideal target for the development of a diagnostic kit for detecting anti-PRRSV antibodies (11). To date, the main commercial ELISA kits for detecting anti-PRRSV antibodies in pig sera are developed with indirect ELISA (iELISA) using PRRSV-N protein as a coated antigen (13) and goat anti-pig IgG as the second antibody. The assays were universally used to be diagnosis of PRRSV infection and evaluation after vaccination. However, this method requires a higher purity antigen and an enzyme-labeled secondary antibody, resulting in a large production cost of the commercial kit.

Nowadays, conventional polyclonal and monoclonal antibodies are widely used as indispensable reagents for the development of disease diagnostic kits (14). Nevertheless, the traditional antibodies have some shortcomings that limit their application in related fields. For example, polyclonal antibodies suffer from batch-to-batch variability, while monoclonal antibodies have high costs and difficult genetic manipulation for production. Thus, there is an urgent need to develop strategies aimed at the production of alternative scaffolds (15). In recent years, single-domain antibodies (sdAbs), also known as nanobodies, are derived from the heavy chain antibody variable region (VHH) in camelids and have attracted much attention in disease diagnosis and treatment (16). Compared with traditional antibodies, nanobodies exhibit more attractive features for diagnostic application, such as small volume (15 kDa), easy genetic manipulation, and high stability (17, 18). Recently, nanobodies have been fused with horseradish peroxidase (HRP) for the development of competitive ELISA (cELISA) to detect antibodies against some animal disease viruses (19, 20). However, the production of the nanobody-HRP fusion protein needs to transfect the HEK293T cell with the plasmid each time, which impedes mass production of the diagnostic kit using the nanobody-HRP fusion protein as reagents.

**FIG 1:**
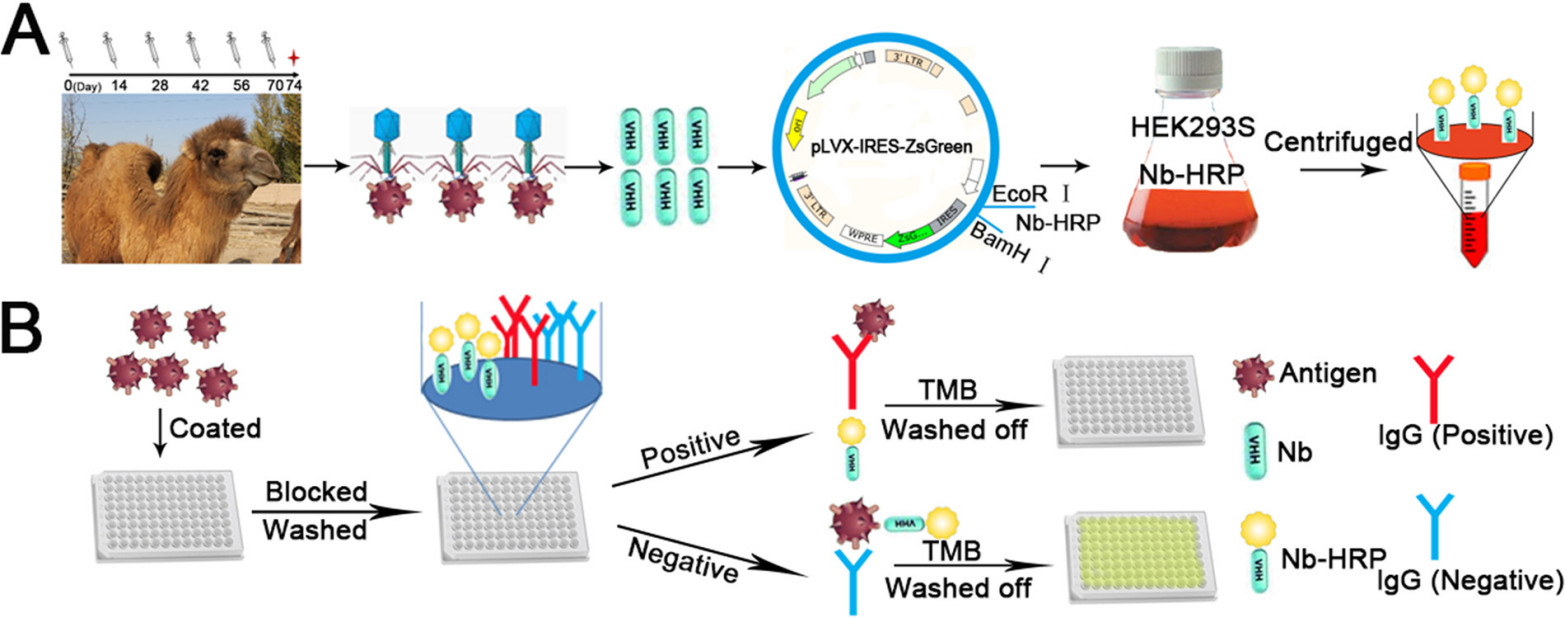
Schematic representation of developing the cELISA to detect PRRSV antibodies using the nanobody-HRP fusion proteins as a reagent. (A) The platform for stably expressing nanobody-HRP fusion proteins using HEK293S cells. (B) Competitive ELISA for using the fusion protein as a reagent.

In the present study, the specific nanobodies against PRRSV-N protein were screened and isolated. Based on the nanobodies, a simple and fast platform for synthesizing nanobody-HRP fusion proteins was developed (Fig. 1A). Then, using the nanobody-HRP fusion proteins as a reagent, a cELISA was developed for detecting anti-genotype 2 PRRSV antibodies in pig sera (Fig. 1B). The developed nanobody-based cELISA showed advantages of simple operation and low-cost production of nanobody-HRP fusion proteins and good sensitivity, specificity, and reproducibility. In addition, the cELISA showed high agreement with the commercial IDEXX ELISA kit and more sensitivity than the kit by detecting the sequential sera from the challenged pigs. Thus, we think that the developed nanobody-based cELISA for detecting anti-genotype 2 PRRSV antibodies was an ideal assay to investigate PRRSV infection in pigs of China and North American and to evaluate the effect of vaccine immunization.

## MATERIALS AND METHODS

### Ethics statement.

The animal studies were carried out in strict accordance with the recommendations in the *Guide for the Care and Use of Laboratory Animals of the Northwest Agriculture and Forestry University* (NWAFU). The animal protocols were approved by the IACUC of NWAFU (20190017/08).

### Cells, viruses, and sera.

Human embryonic kidney cells (HEK293T) and African green monkey kidney cells (MARC-145) were cultured in Dulbecco’s Modified Eagle’s Medium (DMEM; Life Technologies Corp, Grand Island, NY, USA) supplemented with 10% fetal bovine serum (FBS, Gibco, Carlsbad, CA, USA) and 1% antibiotic-antimycotic (Life Technologies Corp.) at 37°C under 5% CO_2_. HEK293S cells were cultured in the commercial serum-free medium (BasalMedia, Shanghai, China) supplemented with 1% antibiotic-antimycotic (Life Technologies Corp.) at 37°C and 130 rpm under 5% CO_2_.

Genotype 2 PRRSV strains SD16 (GenBank ID: JX087437) and NADC30-like (GenBank ID: KX766379) and genotype 1 strain GZ11-G1 (GenBank ID: KF001144) were propagated and titrated in MARC-145 cells in DMEM supplemented with 3% FBS.

The 217 negative sera for anti-PRRSV antibodies were obtained from the healthy pigs and were verified to be negative via a commercial ELISA kit (IDEXX, Westbrook, ME, USA). Serum samples from the pigs challenged with PRRSV HuN4, SD16, and CH-1R strains were used to evaluate the cELISA assay (21–23). To determine the agreement rate of cELISA, 381 challenged sera samples were collected from our previous animal experiments (24, 25). Meanwhile, 450 clinical sera samples were collected from various farms in Shandong and tested using both the commercial ELISA kit and cELISA.

### Animal experiments.

Nine 4-week-old piglets were obtained from a PRRS-free farm and tested for negative PRRSV via the detection of real-time RT-PCR and anti-PRRSV antibodies (26, 27). The piglets were randomly divided into three groups (3 pigs per group), which were separately raised in different isolation rooms. Group 1 piglets were intranasally administered with 1 × 10^6.5^ TCID_50_ of PRRSV NADC30-like strain, group 2 with 2 × 10^5^ TCID_50_ of PRRSV GZ11-G1 strain, and group 3 with 2 ml MARC-145 cell culture supernatant as the negative control. Serum samples were collected at 0, 1, 3, 5, 7, 10, 14, 21 and 28 days postinoculation (dpi) then used for the detection of antibodies against PRRSV-N protein by cELISA and a commercial IDEXX ELISA kit.

### Bactrian camel immunization and library construction.

A 4-year-old male Bactrian camel was immunized six times subcutaneously with the 2 ml killed PRRSV (CH1a strain, 10^5.3^ TCID_50_/ml) (28). Every 2 weeks, the camel was immunized once, and after six immunizations, a serum sample from the Bactrian camel was collected and tested for anti-PRRSV antibodies using iELISA. Four days after the last immunization, peripheral blood mononuclear cells (PBMCs) were isolated from 200 ml blood sample by Leucosep tubes (Greiner Bio-One, Frickenhausen, Germany). Total RNA was extracted from the 1 × 10^7^ PBMCs and reverse transcribed into cDNA. Then, the Camelidae heavy chain-only antibodies (VHH) genes were amplified using the cDNA as a template by nested PCR, as described previously (29). The first PCR products (∼700 bp) amplified with the CALL001 and CALL002 primers (Table 1) were purified according to the instructions of the EasyPure Quick Gel extraction kit (TransGen Biotech, Beijing, China). The second PCR with primers VHH-FOR and VHH-REV (Table 1) was amplified using the first purified PCR products as the template. The final purified PCR products (∼400 bp) were ligated into the phagemid vector pMECS with *Not* I and *Pst* I enzymes sites by T4 DNA ligase (NEB, Ipswich, MA, USA). Then, the ligation products were electro-transformed into competent E. coli TG1 cells. Cells were cultured on LB agar plates with 2% glucose and 100 μg/ml ampicillin at 37°C overnight. Subsequently, the bacterial colonies were scraped from the plates, re-suspended in phosphate buffer saline (0.01 M PBS, pH 7.2) to prepare the VHH phage library against PRRSV, and stored at −80°C.

**TABLE 1:** Primers used in this study^*a*^

Primers	Sequence (5′- 3′)	Usage
CALL001	GTCCTGGCTGCTCTTCTACAAGG	Overlap-VHH
CALL002	GGTACGTGCTGTTGAACTGTTCC	
VHH-FOR	CAGGTGCAG*CTGCAG*GAGTCTGGGGGAGR	
VHH-REV	CTAGT*GCGGCCGC*TGAGGAGACGGTGACCTGGGT	
PRRSV-N-F	*CGCGGATCCATGCCAAATAACAACGGCAAGC*	pET28a-PRRSV-N
PRRSV-N-R	*CCCAAGCTTTCATGCTGAGGGTGATGCTGTG*	
Nb-F	AA*CTGCAG*ATGGAGACCGACACC	pCMV-N1-Nbs-HRP
Nb-R	ATAAGAAT*GCGGCCGC*TTAGTGGTGATGGTG	

*^a^* Restriction sites are underlined.

### Expression and purification of recombinant PRRSV capsid protein.

The ORF7 gene encoding the PRRSV-N protein was amplified using an infectious PRRSV cDNA clone pBAC-SD16 as the template (30). The PCR products were purified and cloned into the pET-28a prokaryotic expression vector (Novagen, Darmstadt, Germany). After sequencing, the recombinant positive plasmid was named pET28a-N. The primers of PCR amplification are listed in Table 1. After the pET28a-N plasmids were transformed into E. coli BL21(DE3) (TransGen Biotech, Beijing, China), the recombinant PRRSV-N protein was expressed and purified based on the previous descriptions (31). Briefly, the positive bacteria were induced with 0.1 mM isopropyl-β-thiogalactopyranoside (IPTG) for 6 h at 37°C. The bacteria were collected and re-suspended in Buffer A (20 mM Tris, 300 mM NaCl, pH 7.2–7.4). The supernatant of bacterial solution was collected after the induced bacteria were ultrasonicated and centrifuged. Subsequently, the supernatant containing recombinant PRRSV-N protein was purified using an Ni-NTA column (Roche, Mannheim, Germany) and eluted with Buffer B (20 mM Tris, 300 mM NaCl, and 250 mM imidazole, pH 7.2–7.4). Finally, the expression, purification, and antigenicity of the recombinant PRRSV-N protein were analyzed by SDS-PAGE and Western blot with the positive pig sera for PRRSV.

### Screening and identification of PRRSV-N specific nanobodies.

The PRRSV-N protein specific nanobodies were screened by three rounds of panning using phage display technology, as previously described, with the following modifications (28). Briefly, the VHH phage library was rescued via M13K07 helper phage. The 96-well plates (Nunc, Denmark) were coated with the recombinant PRRSV-N protein (4 μg/well) overnight at 4°C for the three rounds of panning. The coated plates were blocked with 200 μl of 2.5% skim milk at 37°C for 1 h and washed with 0.05% PBS’T (1 L PBS with 0.5 ml Tween 20). Then, the above rescued recombinant phage (5 × 10^11^ PFU/ml) were added to the plates and incubated at room temperature (RT) for 1 h. After the plates were washed again, the binding phages were eluted using 100 μl 0.1 M trimethylamine (Sigma, St. Louis, MO, USA) and neutralized with same volume of 1 M Tris-HCl (pH 7.4). Subsequently, the growth log phase of E. coli TG1 was infected with the eluted phages and amplified for further rounds of selection. The enrichment of specific phage particles was analyzed using anti-M13/HRP conjugate ELISA combined with phage titration after three rounds of panning. Finally, the 96 colonies were picked randomly and induced with IPTG (1 mM) to express soluble VHHs with an HA-Tag. These recombinant VHHs-HA-Tag proteins were extracted and tested for their capacity to recognize the PRRSV-N protein using iELISA with an anti-HA-Tag antibody as the first antibody (GenScript, Biotech Corp., China). Finally, the positive clones were sequenced, and the nanobodies were grouped according to the hypervariable complementary-determining region 3 (CDR3) sequence.

### Establishment of HEK293S cell lines stable expression of nanobody-HRP fusion protein.

To select the best nanobody to construct the stably expressed cells for producing the nanobody-HRP fusion protein, the different fusion proteins were first expressed with transient transfection. The recombinant plasmids were constructed based on the previous descriptions (19, 32). The VHH gene was amplified using primers Nb-F and Nb-R (Table 1) with pMECS-VHH plasmid as the template. Then, the PCR products and pCMV-N1-HRP vector were both digested via the *Pst* I and *Not* I enzymes and ligated with T4 ligation enzymes to create the recombinant pCMV-N1-Nbs-HRP plasmids. Next, the HEK293T cells were transfected with the recombinant plasmids to produce the nanobody-HRP fusion proteins using polyetherimide (PEI; Polysciences Inc., Warrington, PA. USA) agents. The cell supernatant containing nanobody-HRP fusion proteins was collected after transfection for 60–72 h. The cELISA procedure was used to select the best nanobody, which is described below. The highest percent competitive inhibition (PI) values of the nanobody-HRP fusion protein were selected for constructing the stably expressing cells.

The platform of HEK293S cell line stably expressing the nanobody-HRP fusion proteins was designed as following: The secreting signal sequence, an HA tag, VHH, HRP, and His tag sequence were obtained from the pCMV-N1-Nbs-HRP with the digestion of enzymes EcoR I and BamH I. Then, the gene was ligated into pLVX-IRES-ZsGreen lentivirus vector digested with the same two enzymes. To produce lentivirus particles, the HEK293T cells were co-transfected with pLVX-IRES-ZsGreen-Nb-HRP, psPAX2, and pMD2.0G plasmids using X-tremeGENE HP DNA transfection reagent (Roche, Basel, Switzerland) according to the manufacturer’s instructions. After transfection for 60 h, the packaging lentivirus was observed under a fluorescence microscope, and the cell culture supernatant was collected. HEK293S cells were transduced using the above recombinant lentiviruses and supplemented with 10 μg/ml of PolyBrene (Sigma, St. Louis, MO, USA). After 48 h, the transduced cells were observed under a fluorescence microscope. Then, HEK293S cells with green fluorescence were sorted by high-speed sorting flow cytometer (BD, USA).

### Biological activity analysis of nanobody-HRP fusion proteins produced by the two systems.

The biological activity (titers and stability) of nanobody-HRP fusion proteins produced by the transient transfection and stable expressing system were compared. First, the titers of the nanobody-HRP fusion proteins from the two systems were tested with direct ELISA using a checkerboard titration. Different amounts of the PRRSV-N protein, i.e., 100, 200, 400, and 800 ng/well, were coated into the 96-well plates, and the different dilution ratios of the nanobody-HRP fusions (1:10, 1:100, 1:500, and 1:1000) were tested. The titer was assessed when the OD_450nm_ value of the direct ELISA was 1.0, and the stability of the fusion proteins produced by the two systems was also evaluated. The two methods were independently repeated five times for producing the fusion proteins. Then, the fusion proteins from the different production batches were detected using direct ELISA with a 1:100 dilution. Subsequently, the stability of fusion proteins from the stable expression system was tested from the second, forth, sixth, eighth, and tenth generations, and the supernatants from these generations were detected using direct ELISA with dilutions of 1:10, 1:100, and 1:1000. In addition, the operations of the two systems were compared based on the procedures for producing the fusion proteins.

### Development of competitive ELISA using nanobody-HRP fusion proteins for detecting anti-PRRSV antibodies.

The cELISA was developed using the nanobody-HRP fusion proteins as a probe according to a reported procedure (19). First, the optimal amount of antigen and dilution of fusion protein were determined using a checkerboard titration test with direct ELISA. Different amounts of the PRRSV-N protein (100, 200, 400, and 800 ng/well) were coated into the 96-well plates, then the dilution ratios of fusion proteins of 1:10, 1:100, 1:500, and 1:1000 were tested. Finally, the optimal amount of antigen and fusions were selected when the OD_450nm_ value of the direct ELISA was 1.0 and the amount of coated antigen was the lowest. Secondly, the optimal dilution ratio of pig sera was determined. Five separate positive and negative pig sera were diluted at 1:10, 1:20, 1:40, 1:80, and 1:160 and tested with the cELISA. The optimal serum dilution was determined according to the smallest ratio of OD_450nm_ values between the positive and negative sera (P/N). Finally, the times of incubation and color reaction after the addition of tetramethylbenzidine (TMB) were separately optimized. The incubation times of the mixtures containing the nanobody-HRP fusions and the positive or negative sera with coated PRRSV-N protein were tested at 20, 30, and 40 min. After incubation, TMB was added to color and tested after 10, 15, and 20 min. The smallest ratio of OD_450nm_ values between the positive and negative sera was selected as the optimal incubation and colorimetric reaction times.

After optimizing the above conditions, cELISA was performed as follows. (1) The 96-well ELISA plate was coated with the optimal amount of PRRSV-N recombinant protein and incubated overnight at 4°C. (2) The plate was blocked with 200 μl 2.5% (wt/vol) non-fat dry milk in PBS’T at RT for 1 h after washed three times with PBS’T. (3) After washed with PBS’T again, each well was added into 100 μl of testing mixtures containing the optimal dilutions of serum sample and nanobody-HRP fusions in 2.5% (wt/vol) non-fat dry milk, then incubated for optimal times at RT. (4) After the plate was washed again in the same way, TMB (100 μl/well) was added and incubated for optimal times at RT. (5) Finally, 3 M H_2_SO_4_ (50 μl/well) was used to stop the colorimetric reaction, and the OD_450nm_ values were read using an automated ELISA plate reader (Bio-Rad, USA).

### Determination of cut-off value, sensitivity, specificity, and repeatability of the cELISA.

The PI values were calculated with following formula: PI (%) = [1 − (OD_450nm_ value of testing serum sample/OD_450nm_ value of negative sample)] × 100%. The 217 negative pig serum samples for anti-PRRSV antibodies were used to determine the cut-off value. After these sera were detected using the developed cELISA, the cut-off value was calculated by the mean PI of 217 negative serum samples plus 3 standard deviations to ensure 99% confidence for the negative sera samples within this range (33).

The sensitivity of cELISA was evaluated by testing sera from the different dpi of the three challenged NADC30-like PRRSV pigs as well as the 164 PRRSV-clinical positive sera confirmed by the commercial ELISA kit. In addition, double dilutions (from 1:10 to 1:5120) of five positive pig sera for anti-PRRSV antibodies were tested using cELISA to determine the lowest detection dilution.

The specificity of the cross-competing assay was assessed between the nanobody-HRP fusions and antibodies against other swine viruses, including porcine parvovirus (PPV), porcine circovirus type 2 (PCV2), porcine pseudorabies virus (PRV), transmissible gastroenteritis virus (TGEV), porcine epidemic diarrhea virus (PEDV), and swine influenza virus (SIV). Standard positive sera for antibodies to the other swine viruses were confirmed by the commercial ELISA kit. Total 164 PRRSV-clinical negative sera were also tested with the cELISA. To further confirm whether cELISA can detect anti-genotype 1 PRRSV antibodies, the sera from the pigs pre- and postchallenged with GZ11-G1 strain (genotype 1) were tested. Meanwhile, the sera samples from the pigs challenged with HuN4, SD16, and CH-1R strains of genotype 2 PRRSV strains were evaluated to determine whether cELISA can detect antibodies against different genotype 2 PRRSV isolates.

To determine the reproducibility of cELISA, eight separate positive and negative clinical pig serum samples were tested and used to perform the intra-assay and interassay variabilities. The interassay variation (between plates) and intra-assay variation (within a plate) were evaluated by the coefficient of variation (CV). Each sample was tested using three different plates to determine the interassay CV, while three replicates within each plate were used to calculate the intra-assay CV (34).

### Comparisons of competitive ELISA with commercial ELISA kit.

To evaluate the agreement of cELISA with the commercial ELISA kit for clinical applications, 381 serum samples from challenged pigs with PRRSV and 450 clinical pig serum samples were tested with each method and analyzed via SPSS software. Among these sera, the results reveal inconsistencies between the two detection methods for these, thus indirect immunofluorescence assay (IFA) verification was subsequently performed.

### Indirect immunofluorescence assay.

IFA was performed as previously described with the following modifications (31). MARC-145 cells were plated in 24-well plate and infected with PRRSV strain SD16 at an MOI of 0.1. At 24 hpi, the cells were fixed with 70% ice ethanol and blocked using 1% BSA for 1 h at RT. Next, cells were incubated with clinical pig serum samples as the first antibody for 1 h at 37°C. Secondary antibody was Texas Red-labeled goat anti-swine (Jackson ImmunoResearch, West Grove, PA, USA). Nuclei were stained with DAPI. Immunofluorescence was observed using a fluorescence microscope (Leica, Wetzlar, Germany).

### Statistical analysis.

Statistical analysis was performed using GraphPad Prism version 5.0 (GraphPad Software, San Diego, CA, USA). Students’ *t* test and Kappa index values were calculated to estimate the platform for HEK293S cell lines stably expressing nanobody-HRP fusion protein, as well as the agreement between cELISA and the commercial ELISA kit. Repeatability was assessed using CV (CV = SD/mean), where a CV value less than 15% for the intra-plate assay was considered an acceptable repeatability level for the assay. These calculations were performed using SPSS software (version 20).

## RESULTS

### Expression and purification of the recombinant PRRSV-N protein.

**FIG 2:**
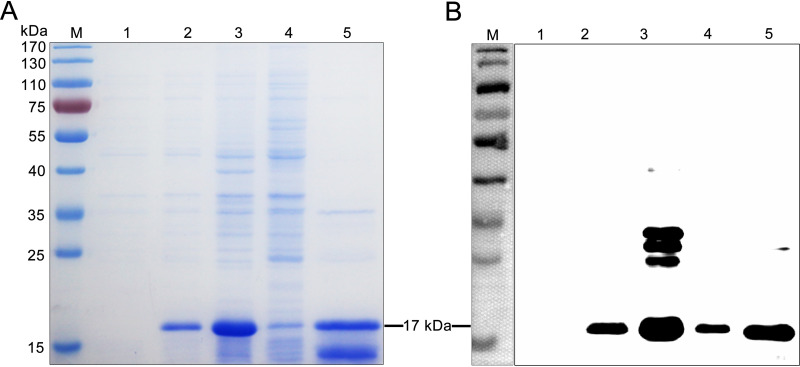
Expression and purification of recombinant PRRSV-N protein. (A) SDS-PAGE analysis of the recombinant PRRSV-N protein. (B) Antigenic analysis of the recombinant PRRSV-N protein using the positive pig sera for PRRSV as first antibody. M: Molecular weight markers; Lane 1: pET28a vector control; Lane 2: Induction with 0.1 mM IPTG; Lane 3: Soluble protein in supernatant after sonication; Lane 4: Inclusion body in precipitation after sonication; Lane 5: Purified PRRSV-N protein.

SDS-PAGE analysis showed that the recombinant PRRSV-N protein was successfully expressed in soluble form with the expected size of 17 kDa (Fig. 2A). In addition, the high purity of the target protein was obtained with the Ni-resin purification (Fig. 2A). To further identify the expression and antigenicity of the target protein, the results of Western blot indicate that the recombinant PRRSV-N protein can react with the positive pig sera for anti-PRRSV antibodies (Fig. 2B). The purified recombinant PRRSV-N protein was used as the coating antigens to screen specific nanobodies and develop the cELISA.

### Screening and identification of anti-PRRSV-N protein specific nanobodies.

A phage display VHH library consisting of approximately 3.2 × 10^8^ individual colonies was constructed from PBMCs of the immunized camel. Positive rate analysis by colony PCR revealed that 96% of these colonies contained a correct insert corresponding to the size of VHH genes. Then, 50 clones were randomly selected, sequenced, and analyzed. The results show that each clone was manifested to contain a distinct VHH sequence (data not shown), suggesting the good diversity and high quality of the library.

**FIG 3:**
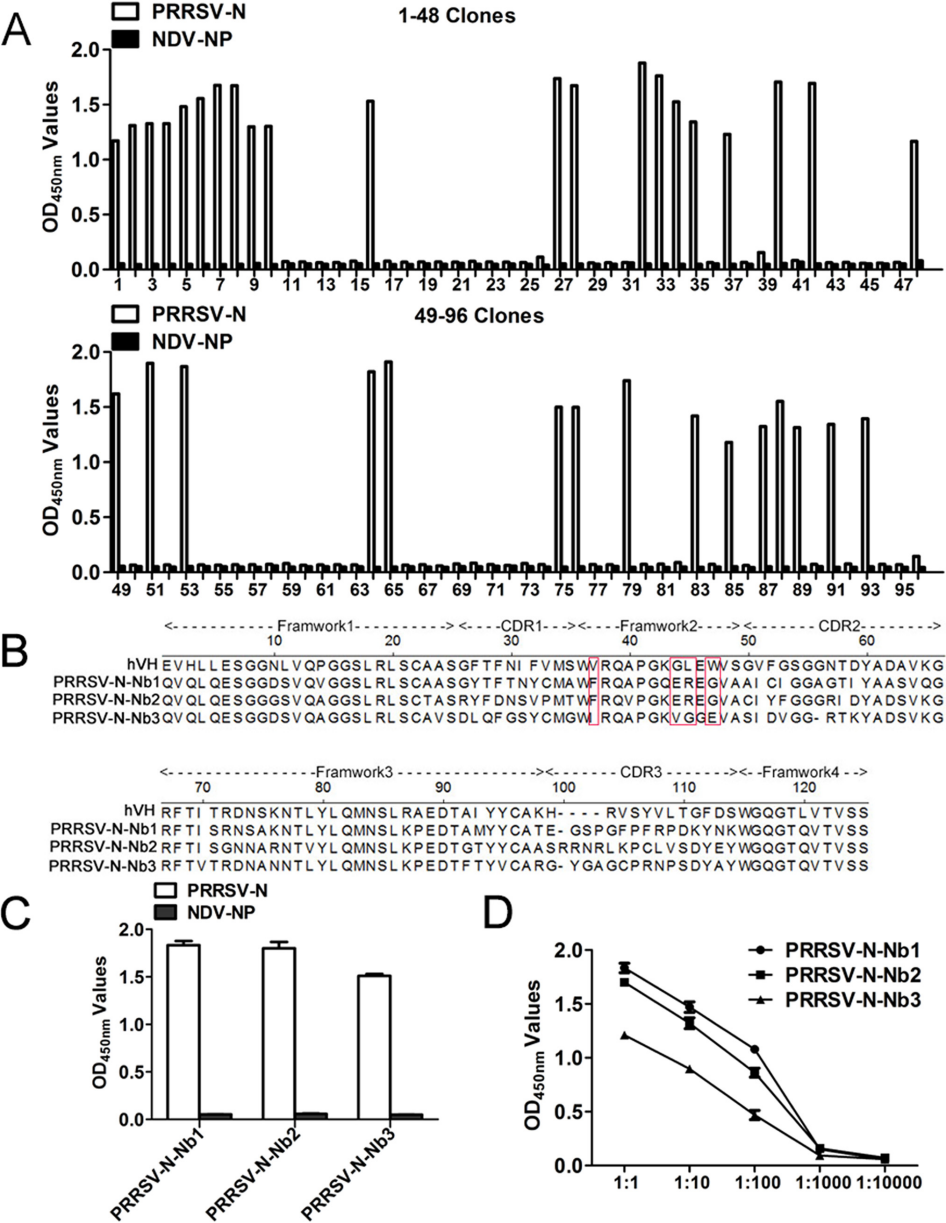
Screening and identification of the nanobodies against the PRRSV-N protein. (A) Identification of the periplasmic extracts from the 96 clones specifically binding to the PRRSV-N protein using iELISA. The 36 clones were positive. (B) Amino acid sequence alignment of three nanobodies against PRRSV-N protein with human VH. The hallmark residues at positions 37, 44, 45, and 47 are highlighted with a red box. (C) Three nanobodies specifically reacted with the PRRSV-N protein using the iELISA. The recombinant NDV-NP protein was used as a negative control. (D) Titration of the nanobodies binding with the PRRSV-N protein in the periplasmic extracts.

After three rounds of biopanning, the specific VHHs phage particles against PRRSV-N protein were enriched (Table 2). Ninety-six mono-clones were randomly selected and expressed periplasmic extracts from the third round of screening for further iELISA detection. The results reveal that 36 individual colonies were identified for specific binding to the PRRSV-N protein (Fig. 3A). Subsequently, the above 36 colonies were sequenced, and three different PRRSV-N specific nanobodies were screened based on the amino acid sequence classification of the CDR3 hypervariable region (named PRRSV-N-Nb1, -Nb2, and -Nb3). The deduced amino acid sequences of the three nanobodies were aligned with the human VH sequence, for which the numbering and CDRs follow the method described by Kabat et al. (35). Alignment results suggest that PRRSV-N-Nb1 and -Nb2 have typical hydrophilic amino acid substitutions in the framework-2 regions Val37, Gly44, Leu45, and Trp47 (located on the VH-VL interface region of VHs) (Fig. 3B). In addition, iELISA results showed that the three nanobodies could specifically bind to recombinant PRRSV-N protein but could not cross-react with the NDV-NP recombinant protein (Fig. 3C). Rather, the recombinant NDV-NP protein was expressed using the same vector pET-28a, which also had a 6×His-Tag, excluding the possibility that these nanobodies might recognize the 6×His region. Moreover, the titers of the periplasmic extracts of PRRSV-N-Nb1 and -Nb2 were higher than that of PRRSV-N-Nb3 (Fig. 3D).

**TABLE 2:** Enrichment of nanobodies against PRRSV-N protein specific phages during three rounds of panning

Round of panning	Phage input (PFU/Well)	Phage output (PFU/Well)	Recovery rate	Enrichment
First round	5 × 10^10^	5.3 × 10^5^	1.06 × 10^−5^	8.5
Second round	5 × 10^10^	1.9 × 10^6^	3.8 × 10^−5^	4.0 × 10^1^
Third round	5 × 10^10^	1.75 × 10^8^	3.5 × 10^−3^	7.0 × 10^3^

### HEK293S cell lines for stable expression of PRRSV-N-Nb1-HRP fusion protein.

**FIG 4:**
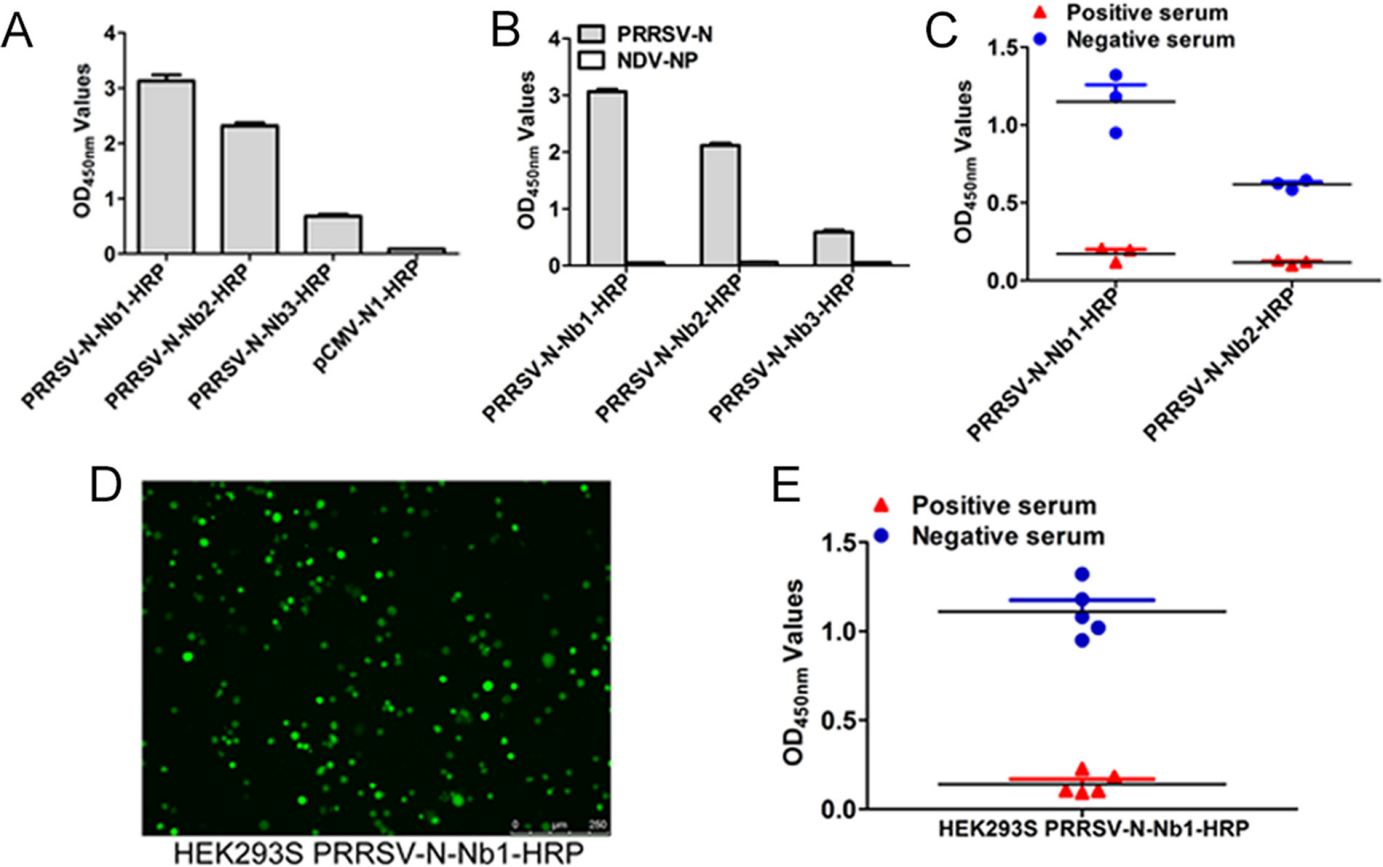
Establishment of HEK293S cell lines for stable expression of PRRSV-N-Nb1-HRP fusion protein. (A) Detection of the binding between PRRSV-N-Nbs-HRP and PRRSV-N using direct ELISA. (B) Specific reactions between PRRSV-N-Nbs-HRP and PRRSV-N using direct ELISA. (C) Comparisons of the two nanobodies blocking the binding between the pig sera and PRRSV-N protein by cELISA. (D) Characterization of HEK293S cell lines for stably expressing PRRSV-N-Nb1-HRP by fluorescence microscopy. (E) Confirmation of the blocking effect of PRRSV-N-Nb1-HRP using the supernatant from the recombinant HEK293S cell lines by cELISA.

For transient expression, the recombinant plasmids, pCMV-Nb1-HRP, -Nb2-HRP, and -Nb3-HRP, were successfully constructed and transfected into the HEK293T cells. An empty vector was used as a control. The results of direct ELISA reveal that the PRRSV-Nb1-HRP, -Nb2-HRP, and -Nb3-HRP fusion proteins were successfully expressed in the form of secretion (Fig. 4A). The three fusions can specifically bind to the PRRSV-N protein (Fig. 4B), indicating that the fusions do not change the antigenic reaction of the nanobody. However, the titers of the supernatant containing the PRRSV-N-Nb1-HRP and -Nb2-HRP fusion proteins were significantly higher than that of PRRSV-N-Nb3-HRP (Fig. 4B). Therefore, the PRRSV-N-Nb1-HRP and -Nb2-HRP fusion proteins were chosen to further evaluate cELISA. Compared with the two fusions blocked by the positive pig sera for anti-PRRSV antibodies to bind the antigen, cELISA results indicate that the blocking rate of PRRSV-N-Nb1-HRP was higher than that of PRRSV-N-Nb2-HRP (Fig. 4C). Therefore, PRRSV-N-Nb1 was selected to construct the stably expressed cells for producing the PRRSV-N-Nb1-HRP fusion protein and for further development of cELISA.

In order to conveniently and quickly produce fusion proteins, the HEK293S cell lines stably expressing PRRSV-N-Nb1-HRP fusion proteins were successfully established. The positive recombinant HEK293S cells were observed under a fluorescence microscope (Fig. 4D), then the cell supernatant was collected and analyzed for the antigenic activity of PRRSV-N-Nb1-HRP fusion proteins using cELISA. As shown in Fig. 4e, the stably expressed fusion proteins from the HEK293S cells can be still blocked to bind the antigens by the positive sera, which is consistent with the expression by transient transfection.

### Comparisons of the two platforms of transient transfection and stable expression.

**FIG 5:**
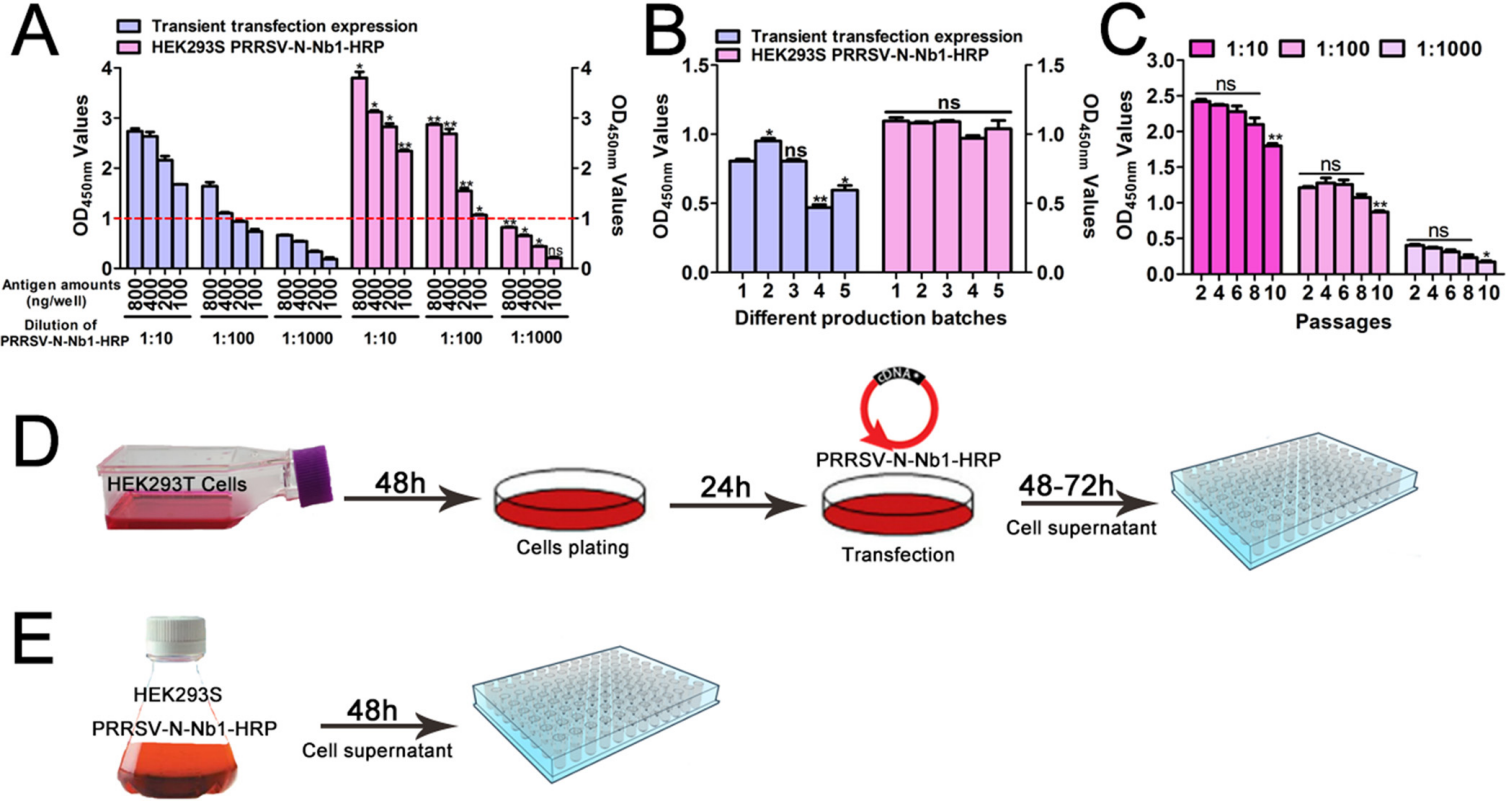
Bioactivity analysis of the PRRSV-Nb1-HRP fusion protein from the stably expressing HEK293S cell lines. (A) Expression level of PRRSV-N-Nb1-HRP fusion protein between stable expression in HEK293S and transient transfection in HEK293T cells. (B) Stability of PRRSV-N-Nb1-HRP fusion protein from the two systems. (C) Stability of the PRRSV-N-Nb1-HRP fusion protein from the different generations of stably expressing HEK293S cell lines. (D) Schematic diagram of the production for PRRSV-N-Nb1-HRP fusion proteins using transient transfection in HEK293T cells. (E) Schematic diagram of the production for PRRSV-N-Nb1-HRP fusion proteins using stable expression in HEK293S cells.

Compared with the transient transfection system, titers of the PRRSV-N-Nb1-HRP fusion protein from the recombinant HEK293S cell lines were higher (Fig. 5A). In addition, with 100 ng/well PRRSV-N protein, the OD_450nm_ value was approximately 1.0 with the dilution of 1:100 for the fusion from stably expressed cells (Fig. 5A). However, the OD_450nm_ value reached 1.0 using coated antigens of 200 ng/well with the same dilution of supernatant from the cells of transient transfection (Fig. 5A). The two systems were independently repeated five times. Although significant differences were observed for the three independent experiments of the transient transfection system, no significant difference was noted for stably expressed cells (Fig. 5B). This suggests a greater stability of the expressed recombinant HEK293S cell lines than the transient system. Moreover, the recombinant HEK293S cell lines for stable expression of PRRSV-N-Nb1-HRP fusion protein could be passed continuously for 8 generations with no difference in titers (Fig. 5C).

In the procedure of the stable expression system producing PRRSV-N-Nb1-HRP fusion proteins, cells were cultured for 48 h then the supernatant was collected for direct use (Fig. 5E). However, the transient transfection system required plating, while plasmids were extracted for each time then transfected into cells. After 48–72 h of transfection, the supernatant was collected for direct use. This production cycle took approximately 132 h (Fig. 5D), and the system required extra costs for the plasmid extraction kit and transfection reagent. The operation procedures of the two platforms indicate that the stably expressed platform is less complex and less costly than the transient transfection system.

### Competitive ELISA using the PRRSV-N-Nb1-HRP fusion proteins as reagents.

The optimal concentration of coated PRRSV-N proteins was determined to be 100 ng/well, and the optimal dilution of PRRSV-N-Nb1-HRP fusion proteins was identified as 1:100 using a checker board titration assay (Table 3). The optimal dilution of the tested pig serum sample was determined as 1:20 based on the different dilutions of 5 positive and negative sera producing the lowest P/N (Table 4). The optimized incubation time of the sera and PRRSV-N-Nb1-HRP fusion protein mixtures was found to be 30 min, and the optimal colorimetric reaction time was 15 min (Table 5).

**TABLE 3:** Optimized amount of PRRSV-N protein as the coating antigen and dilution of PRRSV-N-Nb1-HRP fusion protein using the direct ELISA^*a*^

Nb1-HRP	OD_450_ values after different antigen coating concentration (μg/mL)				
	8.0	4.0	2.0	1.0	0
1:10	3.339	3.206	3.08	2.421	0.051
1:100	2.915	2.653	1.724	1.021	0.081
1:500	1.235	0.987	0.712	0.48	0.072
1:1000	0.686	0.497	0.342	0.187	0.075

*^a^* The optimal amount of PRRSV-N protein and dilution of PRRSV-N-Nb1-HRP were selected when the OD_450nm_ values of the direct ELISA was approximately 1.0.

**TABLE 4:** Optimized dilution of tested pig sera for cELISA^*a*^

Sample no.	Serum type	Dilutions of the pig serum samples				
		1:10	1:20	1:40	1:80	1:160
1	Positive	0.12	0.15	0.24	0.28	0.52
	Negative	1.07	1.14	1.05	1.18	1.23
	P/N	0.11	0.13	0.23	0.24	0.42
2	Positive	0.17	0.21	0.33	0.49	0.87
	Negative	1.06	1.13	1.07	1.09	1.23
	P/N	0.16	0.18	0.31	0.45	0.71
3	Positive	0.14	0.17	0.28	0.37	0.61
	Negative	1.07	1.13	1.18	1.20	1.18
	P/N	0.13	0.15	0.24	0.31	0.52
4	Positive	0.10	0.12	0.22	0.23	0.44
	Negative	1.07	1.13	1.15	1.13	1.15
	P/N	0.10	0.10	0.19	0.20	0.38
5	Positive	0.12	0.15	0.26	0.31	0.53
	Negative	1.10	1.17	1.15	1.15	1.15
	P/N	0.11	0.13	0.22	0.26	0.46

*^a^* Five positive and negative sera were tested using cELISA. The best dilution was selected when the OD_450nm_ values of positive to negative (P/N) sera was smallest.

**TABLE 5:** Optimal competition time of the mixture containing sera and PRRSV-N-Nb1-HRP fusion proteins incubated with the antigen and colorimetric reaction using a checkerboard assay with cELISA^*a*^

Times of color reaction (min)	Sera type	Incubation time (min) of antigens, sera, and PRRSV-Nb1-HRP fusions		
		20	30	40
10	Positive	0.057	0.165	0.173
	Negative	0.103	1.031	1.077
	P/N	0.553	0.160	0.161
15	Positive	0.057	0.168	0.183
	Negative	0.103	1.066	1.103
	P/N	0.553	0.158	0.166
20	Positive	0.058	0.172	0.202
	Negative	0.117	1.068	1.088
	P/N	0.496	0.161	0.186

*^a^* The best competition time and colorimetric reaction time was also selected when the OD_450nm_ values of positive to negative (P/N) sera was smallest.

To determine the cut-off value of cELISA, the results reveal that the average PI (X) value of 217 negative sera was 2.49% with an SD of 6.9%. The cut-off value of cELISA was determined to be 23.19% (2.49% + 3SD). Therefore, the PI of pig serum sample ≥ 23.19% is considered positive, while PI < 23.19% is negative.

### Sensitivity, specificity, and reproducibility of the competitive ELISA.

**FIG 6:**
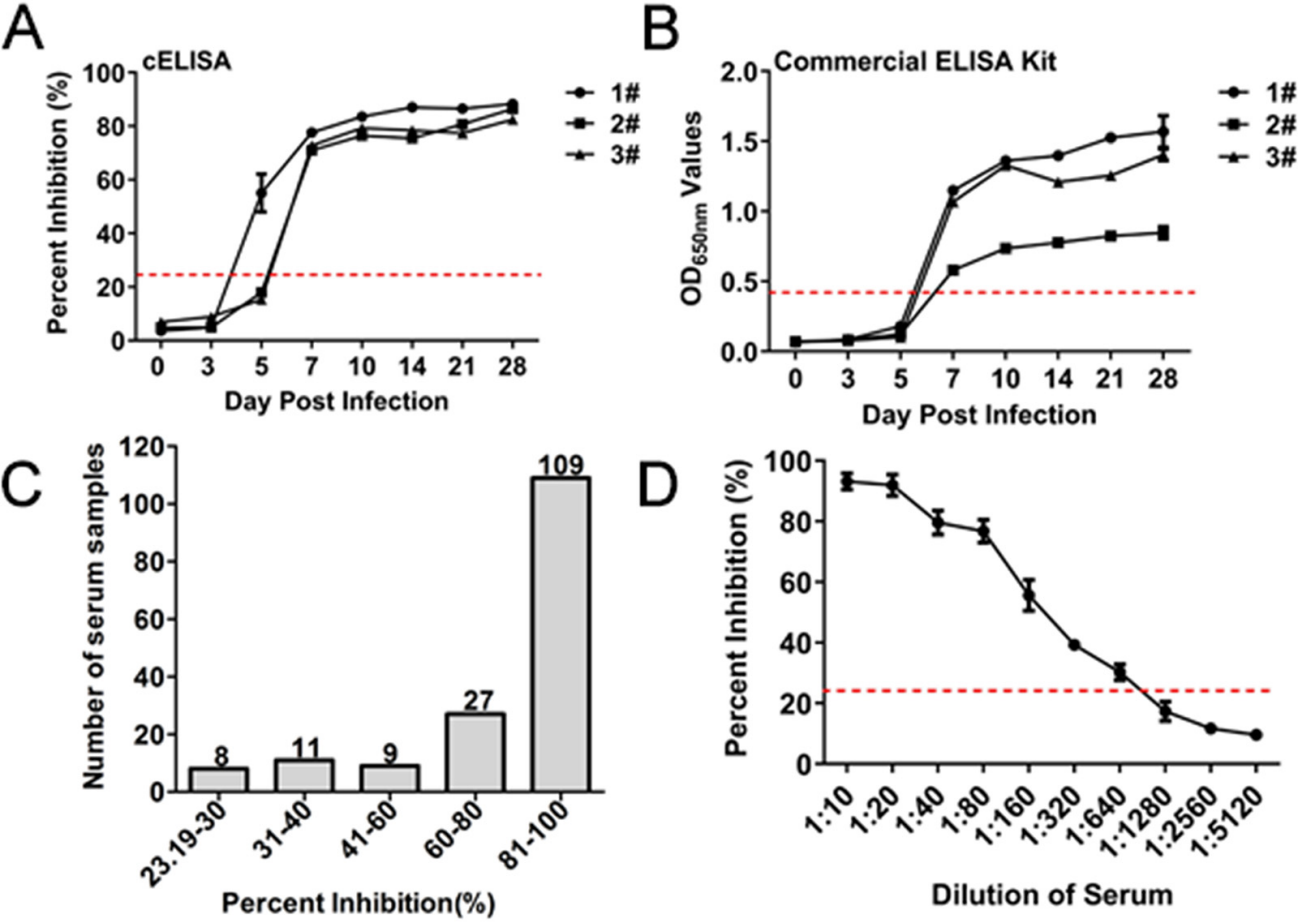
Sensitivity of cELISA using the PRRSV-N-Nb1-HRP fusion protein as a probe. Comparisons of the sensitivity between cELISA (A) and the commercial IDEXX ELISA kit (B) for detecting the sequential serum samples from the three challenged pigs with NADC30-like strain. (C) Distribution of the PI values by detecting the clinical positive sera for anti-PRRSV antibodies using cELISA. (D) Determination of the largest dilution of positive pig sera for anti-PRRSV antibodies.

The sera from the pre- and postchallenged pigs with NADC30-like PRRSV and 164 positive clinical serum samples were tested to assess the sensitivity of cELISA. Seropositivity was first observed at 5 dpi in one of the three pigs, the other two pigs at 7 dpi, which is consistent with the commercial IDEXX ELISA kit. All sera were still positive for anti-PRRSV antibodies until 28 dpi (Fig. 6A). Comparatively, the seropositivity was first observed at 7 dpi in all three pigs with the commercial IDEXX ELISA kit (Fig. 6B). These results suggested that the cELISA has a higher sensitivity than the commercial IDEXX ELISA kit for determining seroconversion of the pigs challenged with PRRSV. For the 164 positive clinical serum samples, the PI values of 109 samples were greater than 80%, and only 8 samples had PI values from 23.19% to 30% (Fig. 6C). These results indicate that the cELISA for testing clinical pig sera being positive for anti-PRRSV antibodies was 100%. For the different dilutions of the 5 positive pig sera using cELISA, sera at a dilution of 1:1280 were negative, and those at 1:320 were positive (Fig. 6D). Similarly, the maximal dilutions of 1:320 for the five positive pig sera were positive with the commercial IDEXX ELISA kit, suggesting that the low limit of the two assays is similar.

**FIG 7:**
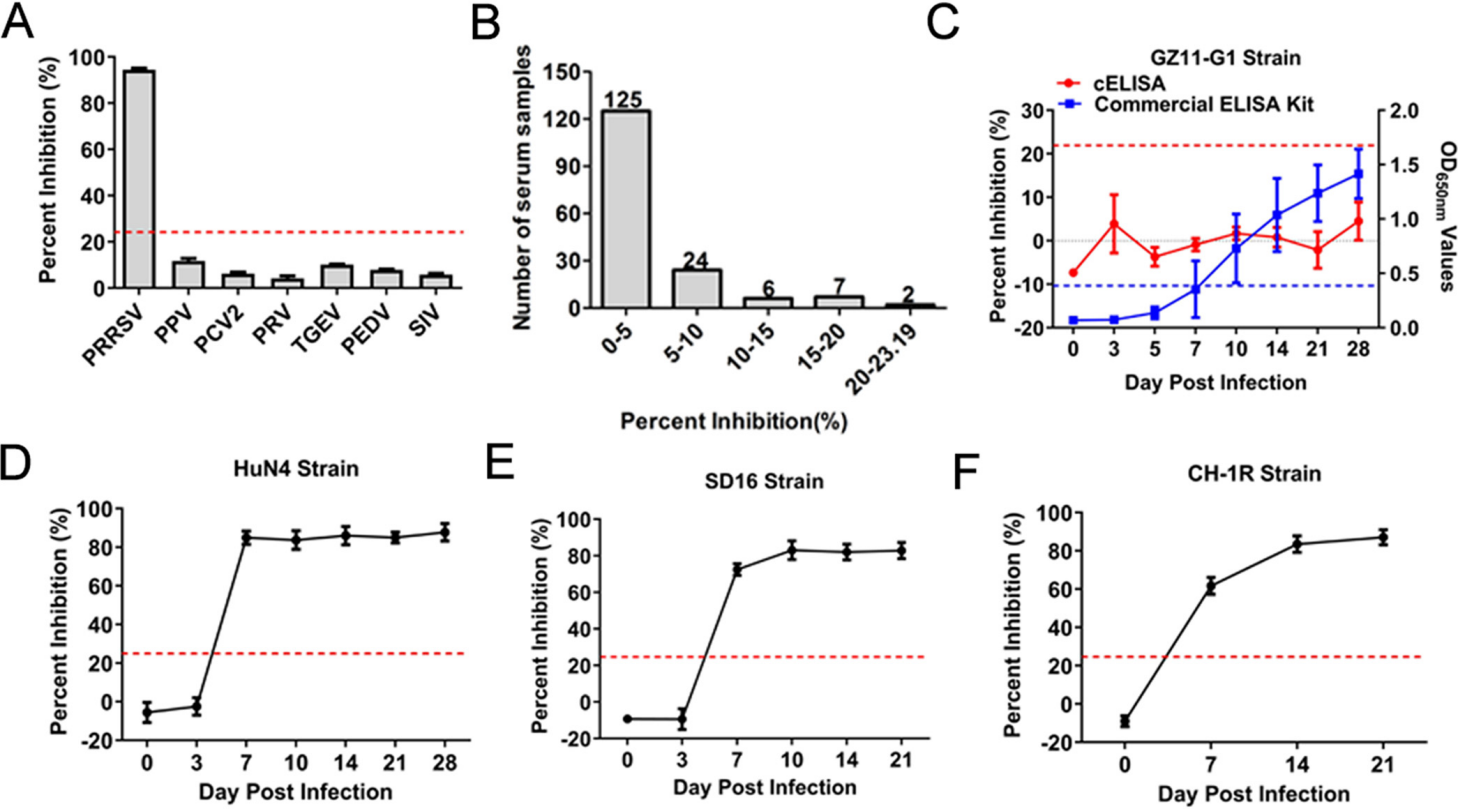
Specificity of cELISA using the PRRSV-N-Nb1-HRP fusion protein as the reagent. (A) Cross-reaction of cELISA by detecting the antibodies against other swine viruses, including PPV, PCV2, PRV, TGEV, PEDV, and SIV. (B) Distribution of the PI values by detecting the clinical negative sera for anti-PRRSV antibodies using cELISA. Pig serum samples were tested using the cELISA from the challenged pigs with different strains of PRRSV, including genotype 1 GZ11-G1 strain (C), HuN4 strain (D), SD16 strain (E), and CH-1R strain (F).

**FIG 8:**
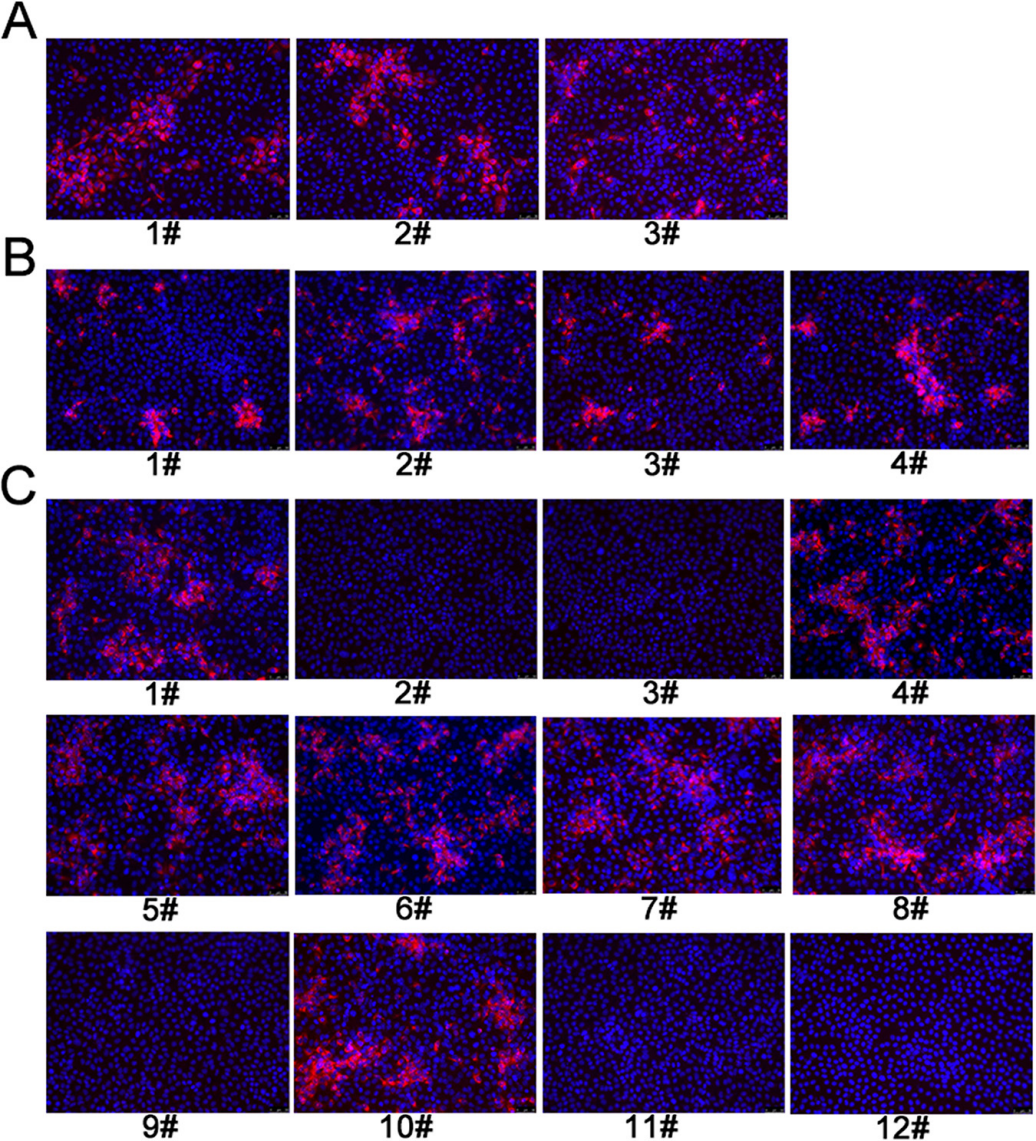
Detection of the pig serum samples with inconsistent results between the developed cELISA and commercial IDEXX ELISA kit by IFA. (A) Three sera positive for cELISA and negative for the commercial IDEXX ELISA kit were tested by IFA. (B) Four clinical pig sera positive for cELISA and negative for the commercial IDEXX ELISA kit were tested by IFA. (C) The remaining 12 sera negative using cELISA and positive using the commercial IDEXX ELISA kit were tested by IFA.

To determine the specificity of cELISA, antisera against other swine viruses, including PPV, PCV2, PRV, TGEV, PEDV, and SIV, were tested using cELISA, using 6 PRRSV positive sera samples as the positive control. According to the results, the PI values of 6 positive serum samples were 79%–91%, while the PI values of antisera against other swine viruses were 1%–19% (Fig. 7A). Furthermore, the 164 negative sera were detected using the cELISA with PI values ranging 1%–20% (Fig. 7B). To further evaluate whether cELISA can test anti-genotype 1 PRRSV antibodies, sera from the pre- and postchallenged pigs with GZ11-G1 strain (genotype 1) were examined. The results revealed that all sera, until 28 dpi, were positive via detection with the commercial IDEXX ELISA kit, but all were negative using the developed cELISA (Fig. 7C). Meanwhile, the sera were also tested from the pre- and postchallenged pigs with HuN4, SD16, and CH-1R strains of genotype 2 PRRSV. Accordingly, seropositivity was first observed at 7 dpi, and all sera remained positive for anti-PRRSV HuN4, SD16, and CH-1R strains of antibodies until 28 or 21 dpi (Fig. 8D to F). Taken together, these results confirm that the developed cELISA can specifically detect anti-genotype 2 PRRSV antibodies.

To analyze the reproducibility of cELISA, eight separate positive and negative clinical serum samples were tested and used to evaluate the intra-assay and interassay variabilities. The intra-assay CV of the PI was analyzed in the range of 0.55%–4.64% with a median value of 2.6%, while the range for the interassay CV was 1.57%–9.53% with a median value of 5.55% (Table 6). These data indicate that the cELISA method exhibits good reproducibility.

**TABLE 6:** Reproducibility of the cELISA determined by intra- and interassay CV value^*a*^

Type of precision	CV (%) value range of 8 serum samples	Median value
Intra-assay precision	0.55–4.65	2.6
Interassay precision	1.57–9.53	5.55

a Intra-assay precision was determined from three repetitions (well-to-well) of 8 serum samples in the same method. Interassay precision was determined from three repetitions (plate-to-plate) at different times.

### Agreements of competitive ELISA and commercial IDEXX ELISA kit.

To evaluate the clinical applications of cELISA, 381 pig sera from the challenged pigs at different dpi (0–28 dpi) were tested with both cELISA and a commercial IDEXX ELISA kit. The results of both methods coincided in 378 (205+/173–) of the 381 serum samples with an agreement rate of 99.2% (Kappa = 0.98). In addition, for the 450 clinical pig sera collected from various farms in Shandong, an agreement rate of 96.4% (Kappa = 0.82) was determined for the two detection methods (Table 7). Statistical analysis further indicates that cELISA had a high level of agreement with the commercial IDEXX ELISA kit, and no significant differences were observed between cELISA and the commercial IDEXX ELISA kit (Kappa values > 0.4) (Table 7). Next, the sera with inconsistent results between cELISA and IDEXX ELISA kit were further tested by IFA. The results showed that the three sera among 381 challenged sera were positive for IFA (Fig. 8A), agreeing with the results of the cELISA. The 16 sera among 450 clinical sera had inconsistent results between cELISA and IDEXX ELISA kit (Table 7). Among the 16 ones, four serum samples were positive for IFA (Fig. 8B), agreeing with the results of cELISA. Among the remaining 12 sera, seven sera were positive by IFA (Fig. 8C), agreeing with the results of IDEXX ELISA kit. Collectively, these results indicated that the cELISA has a high agreement with the commercial IDEXX ELISA kit and IFA, and it is promising for clinical testing.

**TABLE 7:** Comparisons of the developed cELISA with commercial IDEXX ELISA kit by detecting challenged and clinical pig serum samples

Samples	cELISA	no.	Commercial ELISA kit		Agreement (%)^*a*^	Kappa value^*b*^	Positive rate^*c*^
			+	−			
Challenged sera	+	208 (A)	205 (B)	3	99.2%	0.98	
	−	173 (C)	0	173 (D)			
Clinical sera	+	395 (A)	391 (B)	4	96.4%	0.82	87.8%
	−	55 (C)	12	43 (D)			

*^a^* Agreement (%) = (B+D) / (A+C).

*^b^* The kappa value > 0.4 was regarded as significant difference.

*^c^* Positive rate (%) = A / (A+C).

## DISCUSSION

PRRS is one of the most common and economically-important infectious diseases of swine globally. Clinical signs of PRRS are not characteristic, and sometimes, the course of PRRSV infection is subclinical, thus laboratory detection methods are necessary for diagnosis. At present, ELISA is the most popular method that is also used to monitor the antibody level on a population. Among the available commercial ELISA kits for detecting anti-PRRSV antibodies, the IDEXX PRRS X3 Ab Test is the most widely used and generally recognized as the *de facto* gold standard (36). However, this commercial iELISA kit requires the use of a second antibody and, as such, is expensive for mass clinical application. Comparatively, the cELISA based on the nanobody-HRP fusion protein developed in the present study demonstrated simple and low-cost production. In addition, the sensitivity of cELISA is higher than the commercial ELISA kit. More importantly, the developed cELISA has a high agreement with the IDEXX PRRS X3 Ab Test. These advantages suggest that the developed cELISA has a good prospect of market application and promotion.

In the present study, the developed cELISA can detect the antibodies against different genotype 2 PRRSV isolates but not genotype 1 PRRSV. This suggests that the epitope recognized by PRRSV-N-Nb1 may be a unique epitope of genotype 2 PRRSV N protein. The homology of the amino acid sequence of the genotype 2 PRRSV N protein ranges between 96% and 100% (37), indicating that the assay may detect antibodies against most of the PRRSV genotype 2 strains. To further analyze the epitope from different PRRSV isolates, more experiments will be needed to determine the key amino acids of the epitope. In fact, through expression of different truncated genotype 2 PRRSV N proteins, Western blot and ELISA, the epitope recognized by PRRSV-N-Nb1 was determined and is amino acid 103 to 109 region (data not shown). And then, alignments of the region from different genotype 2 PRRSV isolates in GenBank showed that the epitope is a highly conserved epitope (data not shown). Such study revealed that the antibodies against all the genotype 2 PRRSV isolates can be detected by the developed cELISA. According to our knowledge, the commercial IDEXX ELISA kit can detect antibodies against both genotype 1 and 2 PRRSV. Therefore, utilizing this kit following the developed cELISA may be advantageous for the differential diagnosis of genotype 1 and 2 PRRSV.

The sensitivity and specificity of different ELISA methods can be determined by using antibodies as critical reagents (38). Despite the use of traditional antibodies, including polyclonal and monoclonal antibodies, for developing ELISA (39), these antibodies have high production costs and require enzyme labeling (40). In the latest research, nanobodies have acquired increased attention as the smallest known antigen binding antibody with simple genetic manipulation and, thus, as a promising new generation antibody for diagnostic applications (41, 42). Therefore, nanobody-HRP fusion proteins have been designed and used to develop ELISA for detecting antibodies against different virus. Compared to traditional antibodies for the commercial ELISA kit, the nanobody-HRP fusion protein is simple and inexpensive to produce and does not require purification or enzyme-labeling. In this study, a nanobody-HRP fusion protein against anti-PRRSV-N protein was produced and used for the first time as a probe to develop a cELISA for detecting anti-PRRSV antibodies in pig sera. The procedures were performed according to a previous study but with some modifications (32). In the present study, the HEK293S cell lines were used for stably expressing the nanobody-HRP fusion protein, which avoids the trouble of each transfection. In addition, the titers of nanobody-HRP fusion proteins in the supernatant from the recombinant HEK293S cell lines were found to be higher than those of the transient transfection system. Moreover, produced nanobody-HRP fusion proteins from different batches were stable using the stable expression system. In conclusion, the procedures of the stable expression system are simple and easy for mass production. Meanwhile, as we know, CHO cell lines are widely used in the process of large-scale commercial antibody production. So in the future, the CHO cell lines stably expressing nanobody-HRP fusion protein will be constructed. And the two cell lines will be compared to determine which cell line is more suitable for large-scale production of nanobody-HRP.

Although the developed cELISA has simple operation, low production cost, and good sensitivity and specificity, the construction of this platform is highly complicated. Specifically, it requires the immunization of camels, screening of functional nanobodies, and establishing a cell line stably expressing nanobody-HRP fusion proteins. This series of tasks is complicated and demands very skilled experimenters to operate, despite the cELISA test only taking 45 min to run. In short, the establishment of this platform is complicated. Yet it can be noted that the platform is successfully established, and it becomes very convenient in production and clinical application. In addition, the nanobody-HRP fusion protein can be produced on a large scale. The developed cELISA doesn’t need to use the enzyme-labeled secondary antibody and possesses some properties such as simpler operation, shorter time-consuming, lower cost, and easy clinical application.
